# Comparison of intermittent fasting and voluntary wheel running on physical and cognitive abilities in high-fat diet-induced obese rats

**DOI:** 10.1371/journal.pone.0293415

**Published:** 2023-12-06

**Authors:** Chaya Gopalan, Paige Niepoetter, Carolyn Butts-Wilmsmeyer, Sai Medavaka, Avery Ogle, Sheyenne Daughrity, Elizabeth Hackmann, Saruveish Mogan, Oskar Lenz

**Affiliations:** 1 Department of Applied Health, Southern Illinois University Edwardsville, Edwardsville, IL, United States of America; 2 Department of Nurse Anesthesiology, Southern Illinois University Edwardsville, Edwardsville, IL, United States of America; 3 Department of Biological Sciences, Southern Illinois University Edwardsville, Edwardsville, IL, United States of America; 4 Center for Predictive Analytics, Southern Illinois University Edwardsville, Edwardsville, IL, United States of America; 5 Department of Psychology, Southern Illinois University Edwardsville, Edwardsville, IL, United States of America; Belgrade University Faculty of Medicine, SERBIA

## Abstract

Regular physical activity is a proven routine for weight management in addressing obesity. Another method that has gained attention for its health benefits is intermittent fasting (IF). Physical and cognitive abilities while on these routines are poorly understood in the obese population. Sixty-five male Sprague Dawley rats at 7 weeks of age were subjected to diet-induced obesity by feeding a high-fat diet (HFD) or a standard diet (SD) for 8 weeks, after which behavioral testing was performed to detect any changes in physical and cognitive abilities. Rats from the HFD-fed (now considered obese) and SD-fed groups were then subjected to IF (18-hour fast and 6-hour feeding daily), voluntary wheel running (VWR), or control conditions for 3 weeks before repeating the same behavioral testing protocol. IF resulted in less weight gain (p<0.05) and elevated ketone levels (p<0.05) in both SD and HFD-fed groups. IF improved physical activity when compared to VWR and control animals in both SD and HFD-fed groups (p<0.05) while the VWR group in the SD-fed rats exhibited less physical fatigue compared to IF and controls (p<0.05). Additionally, elevated ketone levels were weakly correlated with decreased physical (p<0.0001) and exploratory behavior (p<0.01). These results suggest that IF is more effective than VWR in HFD and SD-fed rats in minimizing weight gain and retaining physical activity, and ketones may play a part in establishing the reported physical benefits. Exploration of physiological mechanisms between ketones, diet, and exercise will help fight obesity and many associated diseases.

## Introduction

Obesity has become an epidemic in the United States, with surveys performed by the CDC from 2017–2018 reporting that 42.2% of adults and 19.3% of children and adolescents are affected by this condition [1, 2]. Obesity is most commonly caused by a net positive caloric intake from reduced physical activity, overeating, or a combination of these factors [3, 4]. Metabolic disorders, cardiovascular disease, cancer, depression, and anxiety are frequently associated with this condition [5]. Additionally, physical, and mental fatigue have been reported in obese individuals. Physical fatigue is defined as the inability of muscles to maintain a needed level of power during and after physical activity [6]. In contrast, mental fatigue is the impairment of cognitive performance due to reduced mental alertness [7–10].

The many health consequences of obesity have increased the need for intervention, including dietary, pharmacological, and lifestyle modifications. Non-pharmacological methods are the most cost-effective and least invasive for individuals [11]. This study tested two non-pharmacological methods, intermittent fasting (IF) and voluntary wheel running (VWR) in a rodent model. Both regimens have been associated with decreasing weight gain and healthy blood glucose levels [12–14]. Additionally, IF and prolonged physical activity have been reported to increase blood ketone levels [15 Mattson] which may be associated with improved physical and mental performance [16–18]. Previous studies have shown that ketones can provide an efficient and readily available energy source for muscles, sparing glucose, and glycogen [19]. Increased ketone utilization appears to encourage the body to rely more on fat oxidation for energy, preserving glycogen stores and extending the duration of physical performance [20]. Additionally, ketosis may lead to reduced lactic acid production, potentially delaying the onset of muscle fatigue during intense exercise [21]. Ketones can also enhance mitochondrial function, reduce oxidative stress, and promote the production of brain-derived neurotrophic factor (BDNF), which is involved in the growth and connectivity among neurons [22].

While physiological measures such as body weight and glucose levels are commonly assessed, the potential for physical and mental fatigue are not as readily investigated. Fatigue is an important subjective component which could influence whether individuals continue participating in weight-loss methods. Therefore, in addition to physiological measures, the assessment of physical and mental fatigue was undertaken in high-fat diet (HFD)-induced obese male Sprague Dawley rats. This study aimed to

1. Compare body weight, blood glucose, and ketone levels in HFD versus standard diet (SD)-fed rats exposed to IF or VWR.
2. Assess physical and cognitive behaviors in HFD versus SD-fed rats exposed to IF or VWR.
3. Correlate changes in ketone levels to performance during behavioral testing.

It was hypothesized that IF and VWR regimens would decrease weight gain, aid in maintaining normal blood glucose levels and increase ketone levels in HFD- and SD-fed rats; IF and VWR applications would improve physical and cognitive abilities in HFD- and SD-fed rats; and increased blood ketone levels would be associated with such benefits.

Experiments using animal work have advantages over studies with humans since the diet can be strictly regulated in the lab condition compared to the subjects’ willpower to withhold from eating for a given time. Moreover, recruiting volunteers is yet another challenge. Animal studies often precede experiments with human subjects or complement in gaining knowledge on ways to combat obesity.

## Materials and methods

### Animals

The animal research conducted in this study underwent a thorough review and received approval from the Institutional Animal Care and Use Committee (IACUC) at Southern Illinois University Edwardsville. This approval, granted through a formal application process, was provided as written consent.

Sixty-five 7-week-old male Sprague Dawley rats were received from Envigo labs (Indianapolis, IN) in two batches. Animals were housed individually under controlled laboratory conditions (12-hour light/dark cycle with lights on at 7:00 PM and off at 7:00 AM at a room temperature of 24–26°C in solid-bottom cages with aspen chip bedding. Rats were allowed three days to habituate in the facility while being fed rat chow and water *ad libitum*.

### Diet

**Fig 1:**
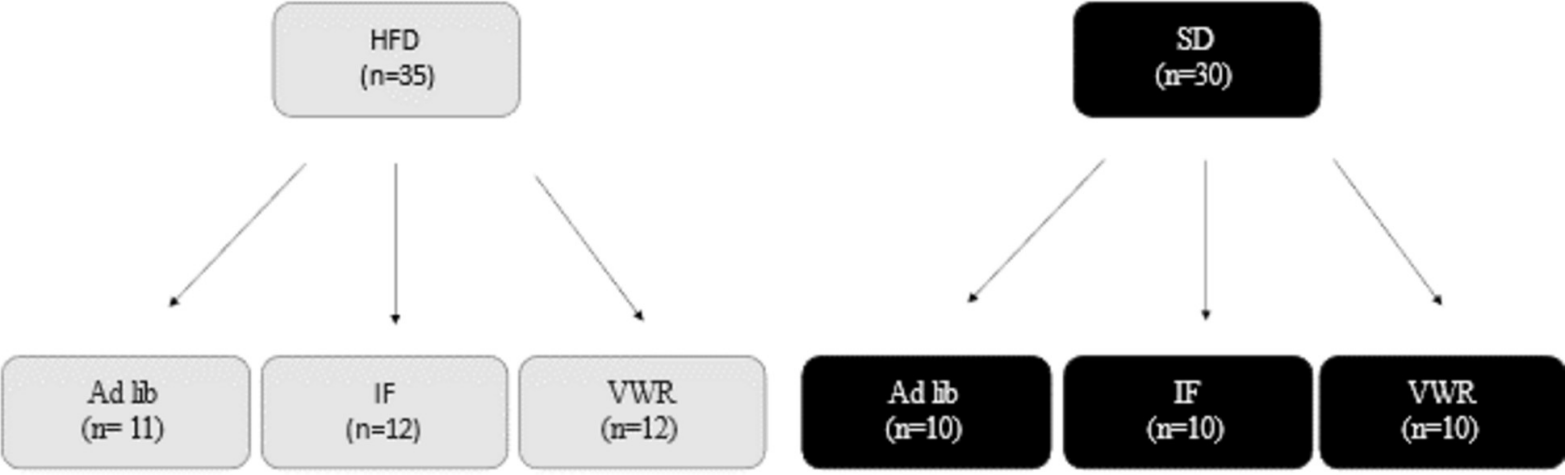
Subdivision of diet groups. HFD: high-fat diet; SD: standard diet; IF: intermittent fasting. VWR: voluntary wheel running.

After habituation, animals were randomly placed into one of the two diet groups (Fig 1): 35 rats were put on a HFD (D12492 from Research Diets Inc; Table 1), and the remaining 30 were put on a SD (D12450J from Research Diets Inc; Table 1). Between weeks 9 and 15, food consumption was measured daily by giving the rats 50 grams of food and measuring the leftover amount after 24 hours.

**Table 1:** Diet composition.

	HFD	SD
Fat (kcal)	60%	10%
Carbohydrate (kcal)	20%	70%
Protein (kcal)	20%	20%
Energy Density (kcal/g)	5.21	3.85
Fat Source	lard, soybean oil	lard, soybean oil

### Metabolic testing

Capillary blood sampling was used to obtain overnight fasting glucose and beta- hydroxybutyrate (BHB) levels between 7:00 and 9:00 AM on day 6 each week (Table 2). Blood samples for this testing were obtained by pricking the rats’ tail veins using 26-gauge lancets. Results were obtained immediately using a *Keto-Mojo* (Napa Valley, CA) glucose and ketone meter (model TD-4279). Previous studies have used capillary sampling and ketone test strips to measure circulating levels of BHB [23, 24].

**Table 2:** Study timeline.

**Phase 1: Induction of Obesity Using HFD**	Weeks 1–8	• 35 rats fed HFD, 30 fed SD. • Blood ketone and glucose testing, body weight monitoring.
	Weeks 9–10	• OF and NOR testing. • Blood ketone and glucose testing, food consumption and body weight monitoring.
**Phase 2: Introduction of Experimental Regimen**	Weeks 11–13	• Rats continue their diets but are divided into control, voluntary wheel running, or intermittent fasting groups. • Blood ketone and glucose testing, food consumption and body weight monitoring.
	Weeks 14–15	• OF and NOR testing. • Blood ketone and glucose testing, food consumption and body weight monitoring.

HFD: high-fat diet; SD: standard diet; OF: open field test; NOR: novel object recognition test; IF: intermittent fasting.

### Intermittent fasting

Rats in the IF group fasted for 18 hours per day and had access to food 6 hours daily between 9:00 AM and 3:00 PM. Fifty grams of food was made available to the rats at 9:00 AM, and the remaining amount was manually collected at 3:00 PM and weighed to measure food consumption. Glucose and ketone levels were measured during the fasting period (7:00 AM -9:00 AM). Rats continued their experimental diets while on IF, and all behavioral testing occurred once the animals had access to food (see Table 2).

### Voluntary wheel running (VWR)

Rats in the VWR group lived in cages equipped with running wheels from Starr Life Sciences. These cages monitored the running activity of the rats 24 hours a day (wheel revolutions per 5 minutes) collected by the *VitalView* software. Researchers have used numerous ways to utilize these measurements. A study by Chowdhury et al. (2021) used wheel rotations to gauge daily voluntary activity [25]. Henriksen and Halseth (1995) also measured running distance daily as they exposed their rats to VWR over the course of 1, 2, or 4 weeks [26]. Corderia and Monahan (2019) allowed rats to access running wheels for 30 minutes daily to gauge voluntary activity and calculated the average distance traveled per day [27]. In this study, the number of revolutions and the diameter of the wheel was used to calculate the distance (m) run each day.

### Behavioral testing: Open field (OF) and novel object recognition (NOR)

In rodent models, locomotor activity and anxiety could be studied using the OF paradigm, while cognition is detected indirectly by measuring recognition memory during NOR testing [28, 29]. OF testing is a commonly used behavioral test that measures exploratory behavior and movement to gauge the physical and mental health of rats and mice. During this test, movements that are recorded consist of line crossings, distance/time moving, rearing, freezing, and defecation [29]. In this study, rats underwent 8 rounds of OF testing both at the end of the DIO phase and at the end of the IF/VWR phase. Each round lasted six minutes. After the completion of OF study, NOR testing was conducted to gauge recognition memory and cognition. NOR is based on the animals’ natural tendency to explore unfamiliar objects [28]. The duration spent investigating the new object vs. the familiar object defines the animal’s recognition of the old object and provides a numerical value to memory. There are many different models of NOR. This study used an intertrial-exposure-interval (IEI) model consisting of habituation, 0-hour, 24-hour, 48-hour, and 72-hours (Table 3). Each round of NOR was 5 minutes long. The habituation phase occurred over 5 minutes with the familiar object, immediately followed by the 0-hour IEI, which contained both the familiar and novel object.

**Table 3:** NOR testing protocol.

Day of NOR	IEI
Day 0	Habituation Phase
	0-hour
Day 1	24-hour
Day 2	48-hour
Day 3	72-hour

### Statistical analysis

Using G*Power 3.1.9.4 and the following parameters (repeated measures ANOVA, effect size f = 0.42, alpha = 0.0167), it was determined that the sample size needed to obtain a power of at least 0.80 was 63 animals. It was hypothesized that the diet will have a medium effect (2 = 0.15). If rats did not gain at least 10% on the HFD relative to the mean of the SD control, these animals were excluded from the analysis, which was accounted for in the G*Power analysis.

In this study, body weight, blood glucose, and blood ketones were measured once per week, and the averages of each of these variables were analyzed using a repeated measures analysis of variance (ANOVA) in PROC MIXED of SAS (version 9.4). A one-way ANOVA with a Bonferroni adjustment was used to analyze the average food and calorie consumption during weeks 14 and 15 in R. Behavioral measurements (i.e., total distance traveled, mean speed, line crossings, and time spent with novel versus familiar objects) were measured on a daily timescale after 3 weeks of IF or the VWR paradigm. Specifically, distance traveled was measured daily for 8 days, and the variables in NOR testing were collected at days 30 (0-hour), 31 (24-hour), 32 (48-hour), and 33 (72-hour) days relative to when the experimental regimens began. As such, repeated measures ANOVA was also used, but the frequency of the repeated measurement with behavioral data was based on a daily model. Additionally, the novel preference was first calculated with the total amount of time spent with the novel object, then as the natural log of the ratio of time spent with the novel object to the time spent with the familiar object. Correlation analysis of ketone levels and behavioral measurements during OF and NOR was performed using the cor.test function in R (version 4.0.4). The significance level was determined by a *p*-value of 0.05.

## Results

### Phase 1: Diet-induced obesity (DIO)

#### Physiological measurements

**Fig 2:**
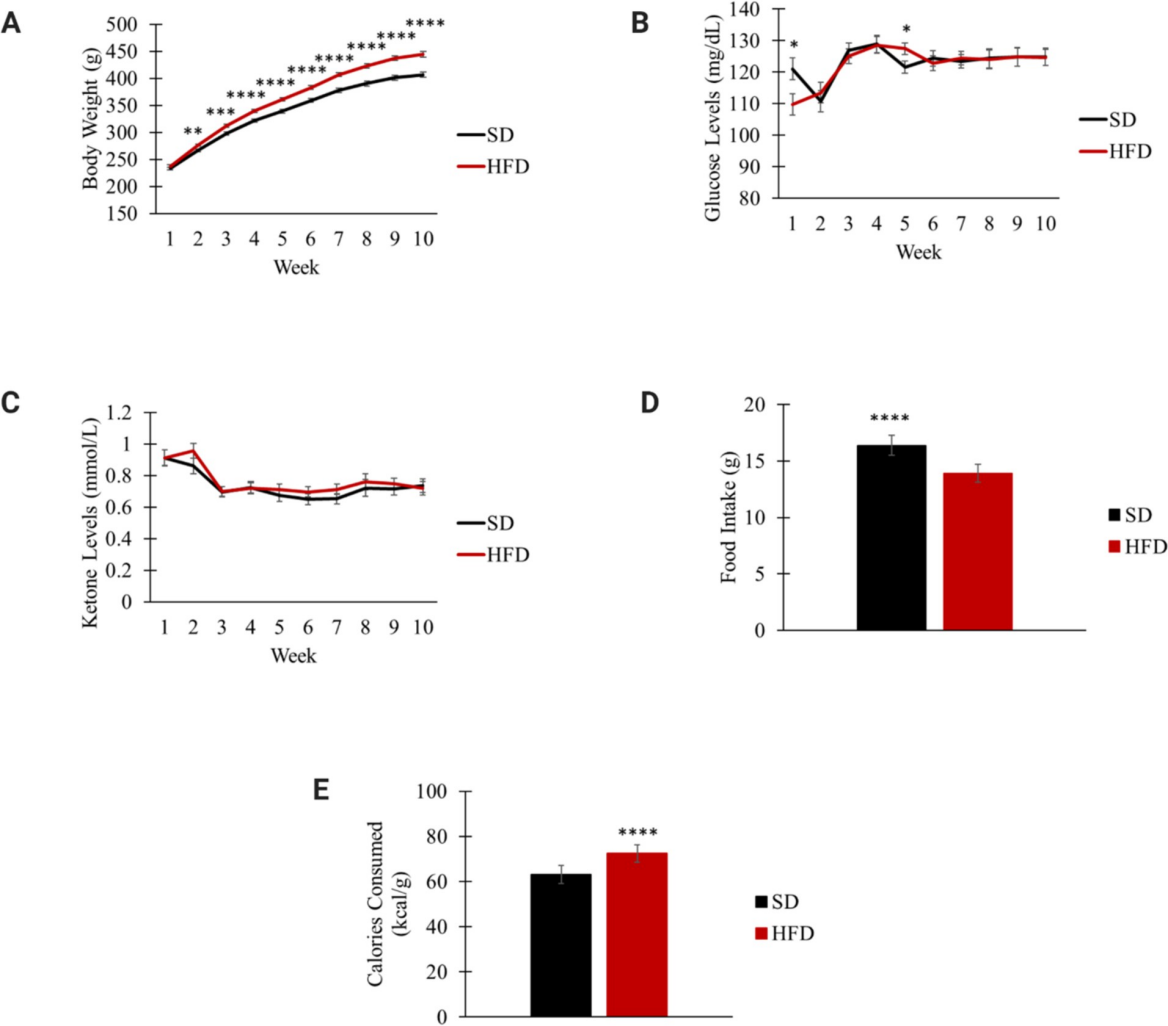
Body weight (A), blood glucose levels (B), blood ketone levels (C), food intake per day (D), and calories consumed (E) during the DIO phase. * p<0.05; ** p<0.01; *** p<0.001; **** p<0.0001 significant differences between SD (n = 30) and HFD (n = 35) groups.

Body weight was significantly different between the HFD (n = 35) and SD (n = 30) groups beginning at week 2. By the end of the DIO phase, HFD-fed rats weighed an average of 30 grams heavier than those fed SD (*p*<0.0001; Fig 2A). Blood glucose and ketone levels did not differ between the HFD and SD groups (Fig 2B and 2C). Rats fed SD ate a greater amount of food versus those fed HFD (SD: 16.4 ± 0.87 g; HFD: 13.9 ± 0.82 g; *p*<0.0001; Fig 2D). However, rats fed HFD had an increased caloric intake compared to those in the SD group (SD: 63.0 ± 4.0 kcal/g; HFD: 72.5 ± 3.8 kcal/g; *p*<0.0001; Fig 2E).

#### Behavioral measurements

**Fig 3:**
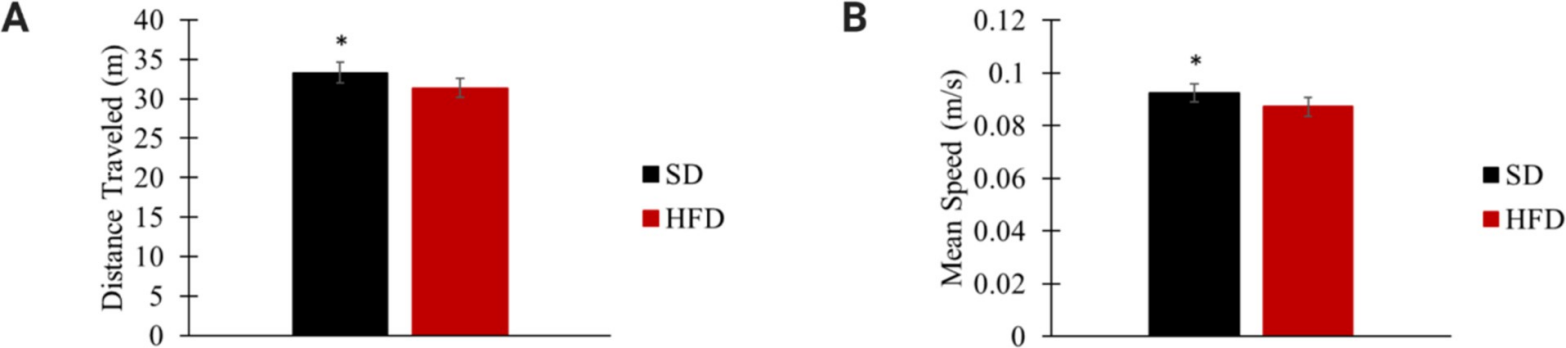
Distance traveled (A) and mean speed (B) during OF testing in DIO phase. * p<0.05 significant differences between SD (n = 30) and HFD (n = 35) groups.

During OF, SD-fed rats ran an overall greater distance than HFD-fed rats (SD: 33.3 ± 1.29 m; HFD: 31.4 ± 1.2 m; *p*<0.05; Fig 3A) and had a greater mean speed (SD: 0.093 ± 0.004 m/s; HFD: 0.087 ± 0.003 m/s; *p*<0.05; Fig 3B). NOR testing did not detect a significant difference in cognition between SD and HFD groups (Table 4).

**Table 4:** Ratio of time spent with novel versus familiar objects during the DIO phase.

IEI	SD	HFD
0-hour	0.72 ± 0.14	0.40 ± 0.12
24-hour	0.02 ±0.08	-0.02 ± 0.07
48-hour	-0.06 ± 0.07	0.10 ± 0.06
72-hour	0.01 ± 0.08	0.28 ± 0.08

Values shown are the natural logs of novel versus familiar objects and the standard errors. IEI: intertrial-exposure-interval. SD (n = 30); HFD (n = 35)

### Phase 2: Experimental regimens

**Fig 4:**
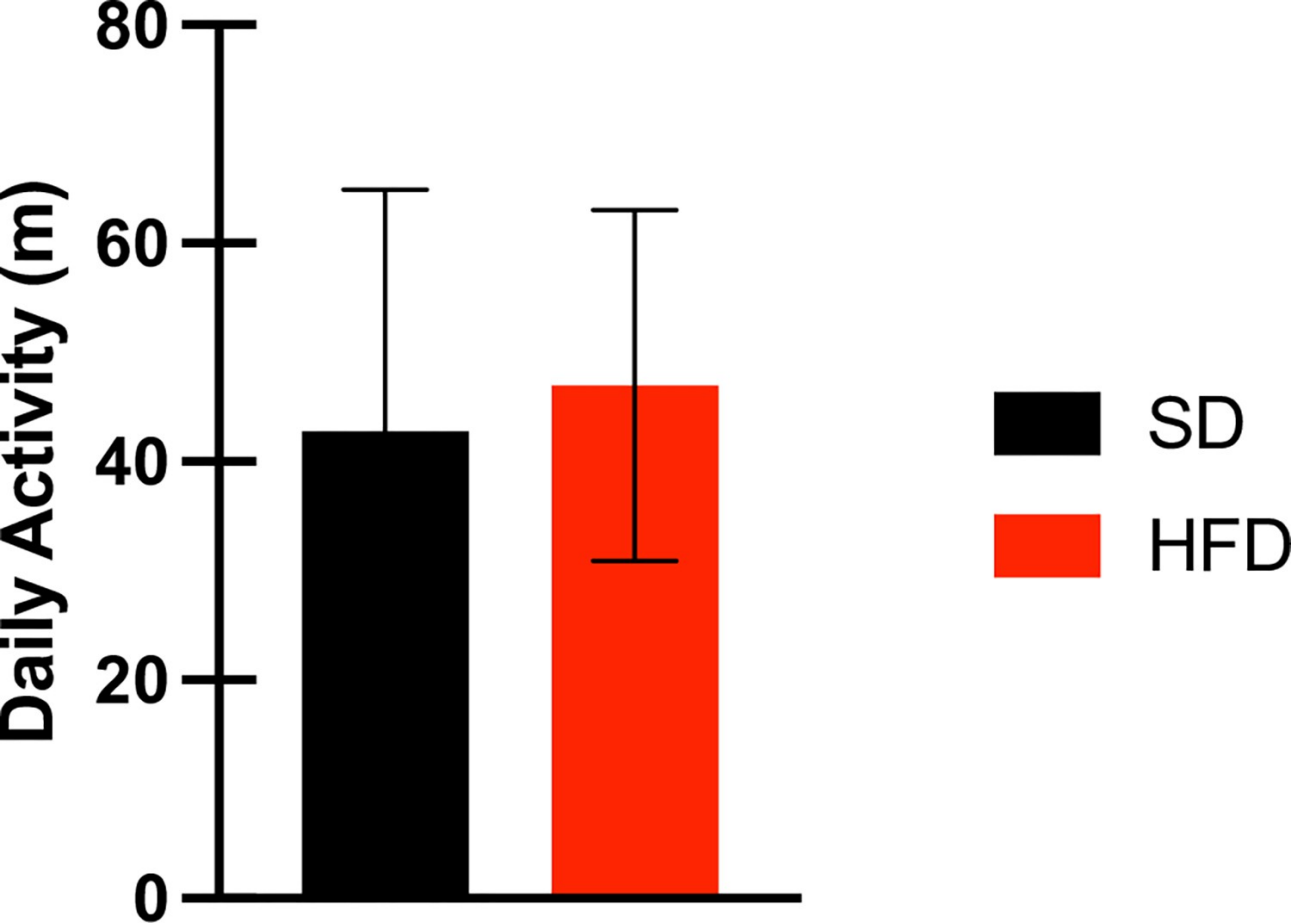
Daily VWR activity in SD (n = 10) and HFD-fed (n = 12) rats.

VWR activity did not differ between SD and HFD-fed rats (SD: 42.76 ± 22.16 m; HFD: 46.96 ± 16.09 m; Fig 4).

#### Physiological measurements

**Fig 5:**
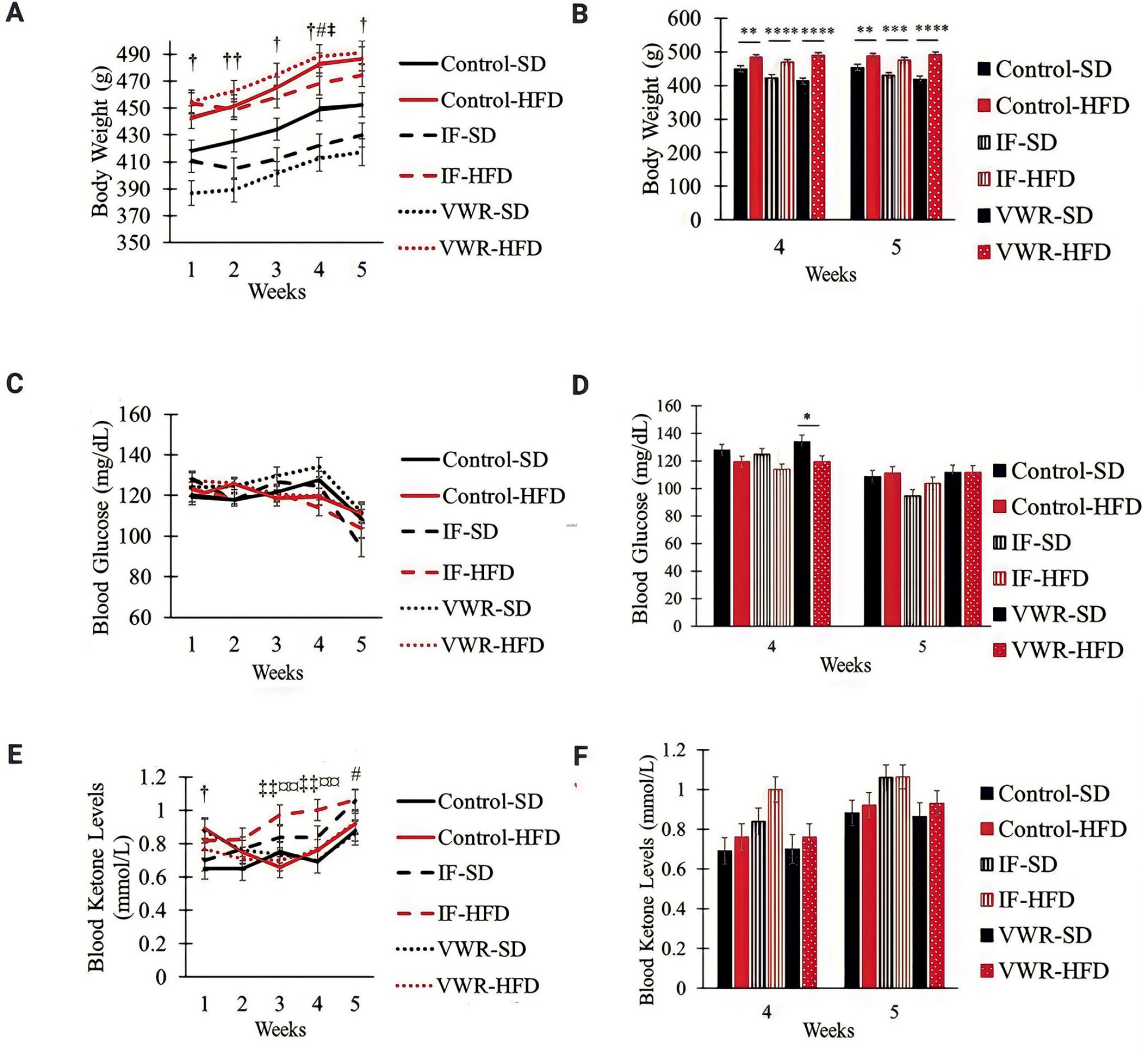
Body weight (A-B), blood glucose (C-D) and blood ketone (E-F) levels in the six experimental groups. † p<0.05 between SD control (n = 10) and VWR (n = 10); †† p<0.01 between SD control and VWR; # p<0.05 between SD control and IF (n = 10); ‡ p<0.05 between HFD IF (n = 12) and VWR (n = 12); ‡‡ p<0.01 between HFD IF and VWR; ¤ p<0.05 between HFD IF and control (n = 11); ¤¤ p<0.01 between HFD IF and control; * p<0.05; ** p<0.01; *** p<0.001; **** p<0.0001 significant differences between SD (n = 30) and HFD (n = 35) groups.

In SD-fed rats, VWR and IF decreased weight gain compared to control animals. This effect became evident at week 4 for both groups (*p*<0.05; Fig 5A). In HFD-fed rats, there was no significant difference in body weight between VWR and control groups. IF, on the other hand, decreased weight gain compared to VWR rats at week 4 (*p*<0.05; Fig 5A). Body weight in the HFD-fed groups was greater than the SD-fed groups (control: *p*<0.01; IF: *p*<0.001; VWR: *p*<0.0001; Fig 5B). There was no significant difference in blood glucose levels between IF, VWR, and control groups in either SD or HFD-fed rats (Fig 5C and 5D). Blood ketone levels were similar between VWR and control groups in SD-fed rats. IF, on the other hand, elevated ketone levels compared to both control and VWR groups in the SD-fed condition (*p*<0.05; Fig 5E). The HFD-fed rats on IF had greater ketone levels than both VWR and control animals (*p*<0.01; Fig 5E). There was no difference in blood ketone levels between VWR and control HFD-fed rats (Fig 5E). No significant differences were detected between HFD and SD-fed rats while on the same experimental regimens (Fig 5F).

#### Food and caloric consumption

**Fig 6:**
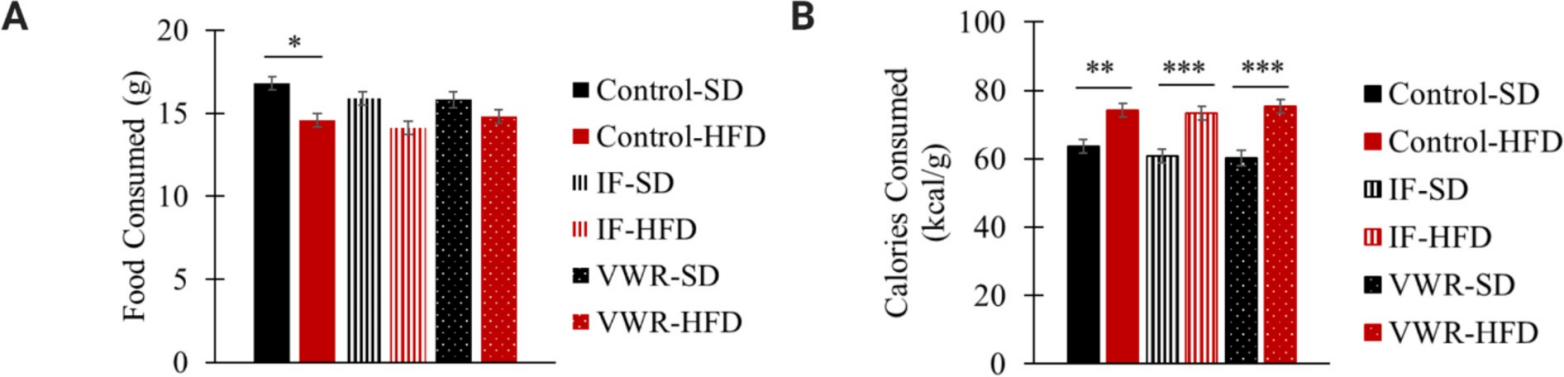
Food (A) and caloric consumption (B) of all six groups. * p<0.05; ** p<0.01; *** p<0.001 significant differences between SD (n = 30) and HFD (n = 35) groups.

Rats fed a HFD consumed similar amounts of food and calories while on IF, VWR, and control conditions (Fig 6A and 6B). SD-fed rats also consumed similar amounts of food and calories while on IF, VWR, and control conditions (Fig 6A and 6B). SD-fed controls consumed a greater amount of food compared to HFD-fed controls (*p*<0.05; Fig 6A). However, rats fed a HFD consumed more calories than those fed an SD in control (*p*<0.01; Fig 6B), IF (*p*<0.001; Fig 6B), and VWR (*p*<0.001; Fig 6B) conditions.

#### Behavioral measurements

**Fig 7:**
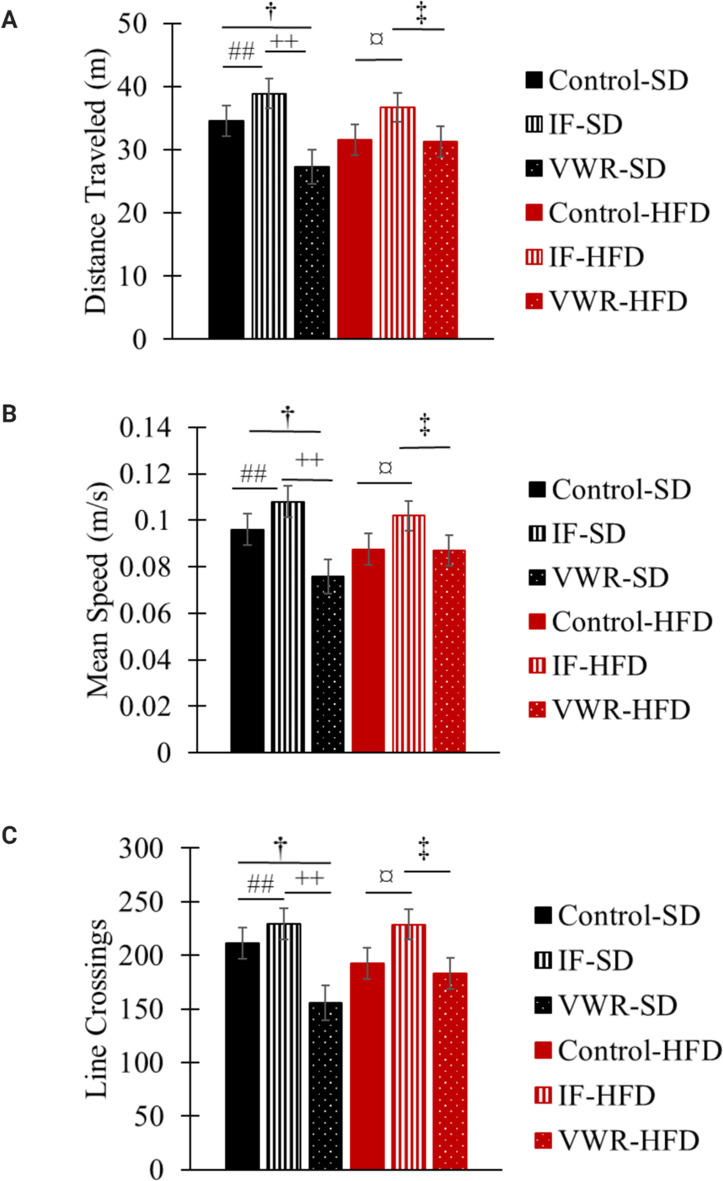
Distance traveled (A), mean speed (B) and number of line crossings (C) by all six groups. † p<0.05 between SD control (n = 10) and VWR (n = 10); # p<0.05 between SD control and IF (n = 10); ## p<0.01 between SD control and IF; ‡ p<0.05 between HFD IF (n = 12) and VWR (n = 12); ++ p<0.01 between SD IF and VWR; ¤ p<0.05 between HFD IF and control (n = 11); ** p<0.01 significant difference between SD (n = 30) and HFD (n = 35) groups.

During OF, IF rats (n = 22) had a greater distance traveled (control: 34.6 ± 2.5 m; IF: 38.9 ± 2.5 m; VWR: 27.2 ± 2.8 m; *p*<0.01; Fig 7A), mean speed (control: 0.10 ± 0.007 m/s; IF: 0.11 ± 0.007 m/s; VWR: 0.08 ± 0.008 m/s; *p*<0.01; Fig 7B), and number of line crossings compared to both control (n = 21) and VWR (n = 22) rats in SD and HFD-fed conditions (control: 211.2 ± 16.1; IF: 229.4 ± 16.1; VWR: 155.7 ± 18.0; *p*<0.01; Fig 7C). VWR rats traveled less distance and had a lower mean speed and number of line crossings compared to control animals in the SD-fed group (*p*<0.05; Fig 7A–7C). VWR rats in the SD-fed group traveled a lesser distance and had lower mean speed and number of line crossings compared to VWR rats in the HFD group (*p*<0.01; Fig 7A–7C). There were no significant differences detected except for the 0-hour measurement during NOR testing, where IF and control rats fed a HFD had better performance than the VWR rats fed a HFD (*p*<0.05; Table 5).

**Table 5:** Ratio of time spent with novel versus familiar objects.

IEI	Control-SD	IF-SD	VWR-SD	Control-HFD	IF-HFD	VWR-HFD
**0-hour**	0.59 ± 0.27	-0.08 ± 0.27	-0.14 ± 0.31	0.54 ±0.15	0.52 ± 0.15	0.06 ± 0.15‡¤
**24-hour**	0.15 ± 0.15	0.09 ± 0.15	0.08 ± 0.17	0.20 ± 0.15	0.19 ± 0.14	0.15 ± 0.14
**48-hour**	0.20 ± 0.18	0.08 ± 0.18	-0.01 ± 0.20	0.19 ± 0.10	0.32 ± 0.09	0.41 ± 0.09
**72-hour**	0.07 ± 0.18	0.05 ± 0.18	0.001 ± 0.21	0.19 ± 0.14	0.19 ± 0.13	0.09 ± 0.13

Values shown are the natural logs of novel versus familiar objects and the standard errors. IEI: intertrial-exposure-interval; ‡ p<0.05 between HFD IF (n = 12) and VWR (n = 12); ¤ p<0.05 between HFD IF and control (n = 11)

A moderately weak positive correlation was found between ketone levels and distance traveled (*p*<0.0001; Table 6), mean speed (*p*<0.0001; Table 6), and line crossings (*p*<0.0001; Table 6). Ketone levels and the ratio of time spent with novel versus familiar objects in all groups were found to have a weak negative correlation (*p*<0.01; Table 6).

**Table 6:** Correlation between ketone levels from all groups and behavioral measurements.

	Distance Traveled (m)	Mean Speed (m/s)	Line Crossings	Ratio of Novel vs. Familiar Object
Correlation	0.27****	0.27****	0.30****	-0.20**

Correlation was determined by the average ketone levels of all rats (n = 65) during the behavioral testing period and the averages of distance traveled, mean speed, line crossings, and ratio of novel versus familiar objects

** p<0.01

**** p<0.0001 significance level

## Discussion

The purpose of this study was to compare body weight, blood glucose, and blood ketone levels in HFD versus SD-fed rats exposed to IF or VWR. Additionally, we assessed physical and mental fatigue during IF and VWR in both HFD and SD-fed animals. A correlation of ketone levels and behavioral factors was also performed to bridge the existing gap in knowledge concerning the role of ketone levels in mitigating the effects of obesity.

We hypothesized that IF and VWR would decrease weight gain, aid in maintaining normal blood glucose levels and increase plasma ketone levels in HFD and SD-fed rats. Prior to investigating the experimental regimens, DIO, as defined by a 10% increase in body weight compared to those fed an SD, was achieved by feeding a HFD over the course of 10 weeks. In both SD and HFD-fed rats, the IF regimen was effective in decreasing weight gain. The IF regimen has been successful for decreased weight gain with many different diets [18, 25, 30]. While IF was effective in both diet groups, VWR was only effective in reducing weight gain in SD-fed rats. Similar findings were reported with a DIO model in 7-week-old C57BL6 mice where a non-significant weight loss was found after exposure to VWR and HFD for 21 weeks [31]. In a study by Cordeira & Monahan (2019), VWR decreased weight in HFD-fed male C57BL/6 mice via decreased food consumption [27]. However, in our study HFD-fed rats on VWR, IF, and control regimens did not differ in food or caloric consumption. To change body weight, physical activity and food intake must lead to a deficit in calories [32]. Therefore, the absence of effect by VWR in the HFD-fed group may be explained by the lack of adequate physical activity needed to mitigate the number of calories being consumed daily.

Throughout the study, blood glucose levels were similar between SD and HFD groups. The similarity in glucose levels between D12492 and D12450J has been attributed to the matching sucrose levels [33–35]. Elks et al. (2015) reported that fasting blood glucose levels in post-menopausal female C57BL/6J mice were similar between groups fed D12492 and D12450J for 12-weeks [33]. This was further supported by Wang et al. (2019) using male and female C57BL/6J mice fed D12492 and D12450J for 4-weeks [35].

IF led to elevated blood ketone levels in both SD and HFD-fed groups in our study. This increase is supported in a study by García-Gaytán et al. (2020), which found that 3-weeks of IF (19-hour daily fast) increased ketone levels (BHB and acetoacetate) in male Wistar rats [36]. Additionally, other studies have found increased ketone levels in as little as one week, as was reported by Rojas-Morales et al. (2020), which used a 22-hour daily fast in male Wistar rats [37]. Since prolonged physical activity has been associated with increased ketone levels, this study investigated if VWR would have similar effects [18, 38, 39]. Ketone levels, however, were not affected by the VWR regimen in either of the diet groups. While studies comparing VWR and IF regimens are scarce, a study by Grossberg et al. (2020) reported similar effects as our study on ketone levels in male C57BL/6J mice. After 25 days of the intervention, it was found that there was an increase in ketone levels induced by IF, but no difference was detected in VWR mice [40]. It is possible that IF was capable of increasing ketone levels due to the stringent adherence forced upon the animal while the completely voluntary nature of VWR and access to food *ad libitum* contributes to the unchanged ketone levels. Alternative models of exercise, such as the forced running treadmill model, have reported increased ketone levels [41, 42]. In such models, the activity level produced is not by choice, thus forcing the animal to utilize more of its glucose stores before initiating the metabolic switch from glucose to fatty acid utilization due to the decrease in carbohydrate availability [19, 43].

Our second hypothesis was that IF and VWR would improve physical and cognitive activities in HFD and SD-fed rats. During OF, IF rats did exhibit greater physical performance compared to VWR and control groups in the form of increased distance traveled, mean speed, and the number of line crossings. Similar results have been reported using male C57BL/6 J mice exposed to an alternate day fasting regimen, where increased distance traveled was observed after 3 weeks on IF [44]. Hazzaa et al. (2020) also reported increased distance traveled during OF using male Wistar rats fed a HFD in an alternate day fasting pattern. The benefits of IF were noted at both 4-weeks and 12 weeks post-exposure to the regimen [18].

VWR, on the other hand, produced less physical activity in the SD group while the HFD group was unaffected. A reduction in physical activity during OF has also been reported by Garrett et al. (2012) using male C57BL/6 J mice on VWR for 28 days [45]. In a study by Hicks et al. (2016), C57BL/6J mice were exposed to VWR for 10-weeks and were fed either a SD or HFD [46]. The mice then underwent OF, where VWR rats traveled a lesser distance and had a decreased velocity compared to control rats in both diet groups (46). Both the current results and previous studies suggest that VWR rats could be less responsive because of access to the running wheel prior to OF testing [45, 47].

There were no differences in performance detected between IF, VWR, and control animals in the SD-fed rats during NOR testing. Similar results were seen in the HFD-fed group, except for the 0-hour time point, where VWR rats spent less time with the novel versus familiar object compared to both controls and IF rats. In a study by Ebada et al. (2016), male C57BL/6J mice were exposed to VWR for 5 weeks prior to NOR testing which showed no significant difference between VWR and sedentary control performance [48]. On the other hand, a study in male sand rats reported improved NOR performance after exposure to VWR for 8 weeks [49]. Bilu et al. (2022) explains that the improvement in cognition is most likely due to increased BDNF levels mediated by VWR activity [49]. If BDNF mediates the cognitive benefits experienced by VWR, it is possible that our study duration of 4 weeks prior to NOR testing was not sufficient for initiating these cellular changes.

To test the hypothesis that increased ketone levels would be associated with improved physical and cognitive performance, a correlation analysis was performed. The correlation analysis showed that higher ketone levels were indeed associated with greater performance during OF, which measures exploratory and physical activity. Similar findings were reported in a study by Murray et al. (2016), which utilized a treadmill test that gradually increased the speed of the treadmill until the rats were fatigued and could no longer maintain the pace of the belt as a measure of physical fatigue. Rats that were fed a 30% ketone diet exhibited higher plasma ketone levels and ran 32% further than control rats [21]. Another study by Brocchi et al. (2022) found that IF increased ketone levels which were associated with increased ATP, decreased reactive oxygen species, and improved mitochondrial function. These systemic changes could have been the cause of an increase in BDNF and fibroblast growth factor 2 concentrations within the brain [49, 50].

While ketones had a positive effect of mitigating physical fatigue in our study, a negative correlation was reported during NOR testing, suggesting that ketones may be associated with impaired cognitive ability. The current findings not only contradict our previous observations [51] but are also at odds with Brownlow et al. (2017) which reports that increased ketone levels were associated with better performance during NOR testing in male Sprague Dawley rats after 3 weeks of receiving a KD [24]. Further investigation is needed to better understand this observation.

A limitation of this study was the short-term duration of experimental regimens compared to those that report beneficial effects when exposed for a longer duration [49, 52, 53]. Both HFD- and SD-fed rats traveled an average of 43 to 47 meters daily, while other studies have reported greater running wheel use [27, 49]. Running wheels were not removed prior to behavioral testing, suggesting that the physical fatigue experienced may be due to the VWR rats engaging in physical activity prior to testing. Given the short-term timeline of the study, it is possible that VWR has the potential to produce the same benefits as IF, but over a longer period. It is likely that IF was able to produce better outcomes in a shorter time due to forced adherence of the regimen, which, in turn, is expected to initiate metabolic changes at a faster rate. Yet another limitation was the use of male rats only in this study. Inclusion of female rats to compare these results is proposed as a future study.

Overall, IF yielded greater outcomes in weight control and resistance to physical fatigue whether maintained on a SD or HFD. Furthermore, it can be elucidated that increased blood ketone levels play a role in physical benefits experienced since IF groups exhibited decreased weight gain and greater physical activity, while VWR did not. While this study aids in bridging the current knowledge gap to some extent, further exploration of these two regimens is needed. An extended exposure to the regimens may increase the metabolic switch from glucose to ketones, which, in turn, could lead to additional behavioral outcomes.
